# Breastfeeding Is Not a Risk Factor for Mother-to-Child Transmission of Hepatitis B Virus

**DOI:** 10.1371/journal.pone.0055303

**Published:** 2013-01-28

**Authors:** Xiangru Chen, Jie Chen, Jian Wen, Chenyu Xu, Shu Zhang, Yi-Hua Zhou, Yali Hu

**Affiliations:** 1 Faculty of Nursing, Nanjing Drum Tower Hospital, Nanjing Medical University, Jiangsu, China; 2 Department of Obstetrics and Gynecology, Nanjing Drum Tower Hospital, Nanjing Medical University, Jiangsu, China; 3 Department of Obstetrics and Gynecology, Zhenjiang Fourth People's Hospital, Jiangsu, China; 4 Departments of Experimental Medicine and Infectious Diseases, Nanjing Drum Tower Hospital, Nanjing University Medical School, Jiangsu, China; 5 Jiangsu Key Laboratory for Molecular Medicine, Nanjing University Medical School, Jiangsu, China; The University of Hong Kong, Hong Kong

## Abstract

**Background:**

Many clinicians do not encourage breastfeeding in hepatitis B virus (HBV) carriers, since HBV DNA can be detected in breast milk and breast lesions may increase exposure of infants to HBV. The aim of this study was to determine whether breastfeeding may add risk for perinatal HBV transmission.

**Methodology/Principal Findings:**

Totally 546 children (1–7-year-old) of 544 HBV-infected mothers were investigated, with 397 breastfed and 149 formula-fed; 137 were born to HBeAg-positive mothers. All children had been vaccinated against hepatitis B but only 53.3% received hepatitis B immune globulin (HBIG). The overall prevalence of HBsAg+, HBsAg−/anti-HBc+, and anti-HBs (≥10 mIU/ml) in children was 2.4%, 3.1%, and 71.6% respectively. The HBsAg prevalence in breast- and formula-fed children was 1.5% and 4.7% respectively (*P* = 0.063); the difference was likely due to the higher mothers' HBeAg-positive rate in formula-fed group (formula-fed 49.0% vs. breastfed 15.9%, *P*<0.001). Further logistic regression analyses showed that breastfeeding was not associated with the HBV infection in the children, adjusting for the effect of maternal HBeAg status and other factors different between the two groups.

**Conclusions/Significance:**

Under the recommended prophylaxis, breastfeeding is not a risk factor for mother-to-child transmission of HBV. Therefore, clinicians should encourage HBV-infected mothers to breastfeed their infants.

## Introduction

Hepatitis B virus (HBV) infection is a global health problem because of its severe sequelae, such as liver cirrhosis and hepatocellular carcinoma. Once infected, the main factor to influence the outcome is the age when the infection occurs; as high as 70–90% of the perinatal infection will become chronic [1]. Thus, prevention in infants as early as possible is critical, especially in those born to HBV-infected mothers. The recommended immunoprophylaxis against hepatitis B, simultaneous use of hepatitis B immune globulin (HBIG) and hepatitis B vaccine in infants of HBV-infected mothers, is most effective to prevent mother-to-child transmission of HBV.

Breast milk is the optimal and natural food for infants. Although World Health Organization and US Advisory Committee on Immunization Practices postulate that chronic HBV infection of the mother could not be an argument against breastfeeding [2], [3], many clinicians do not encourage HBV carrier mothers to breastfeed their babies [4], [5], since HBV DNA can be detected in breast milk [6] and breast lesions such as cracked or bleeding nipples may increase exposure of infants to HBV. Recent studies reported that many HBV-infected mothers tend to formula-feed their infants to prevent mother-to-child transmission of HBV [7]–[9], although there are studies suggesting that breastfeeding does not add risk for the mother-to-child transmission of HBV [10]–[12].

In this study, we compared the HBV infection rates in HBV-infected mothers' children, who were breastfed and formula-fed respectively, to clarify whether breastfeeding may pose additional risk for the mother-to-child transmission of HBV.

## Materials and Methods

### Subjects and serum specimens

In a retrospective study on the prevalence of hepatitis B surface antigen (HBsAg) among 6398 pregnant women, whose serum samples were collected during 2002–2004, 429 (6.71%) women were positive for HBsAg and antibody against hepatitis B core antigen (anti-HBc) [13]. From October 2009 to March 2010, we invited these mothers and their children to participate in the present study. After getting the written informed consent from 296 mothers, a total of 296 serum samples (∼3 ml each) from their 296 children (aged 5–7 years) were collected. In addition, we invited 385 HBsAg-positive pregnant women who delivered their babies in Zhenjiang Fourth People's Hospital from January 2006 to December 2010 to join this study; 248 accepted our invitation and consented their own and their 250 children's blood sampling during November 2011 to March 2012. Therefore, totally 546 children (2 pairs of twins) born to 544 HBsAg-positive mothers were enrolled in our investigation.

Each mother was asked to complete a questionnaire, including demographic information of the mother and her child/children, vaccination against hepatitis B, especially timely first dose vaccine and administration of HBIG in the child. Formula-fed infants were fed exclusively with formula, while breastfed infants were defined as those who received breast milk for at least 2 weeks as previously described [11]; those who received breast milk fewer than 2 weeks were not included in either group. HBV serological markers and HBV DNA in pregnant women were tested or obtained from the hospital chart records. Administration of HBIG and the first dose vaccine was further validated by the hospital discharge records. The injection of second and third doses vaccine was confirmed by children's vaccination records.

This study was performed according to the Declaration of Helsinki and approved by the institutional review boards of Nanjing Drum Tower Hospital and Zhenjiang Fourth People's Hospital. Each mother signed the written informed consent for herself and her child.

### Detection of HBV serologic markers and HBV DNA

All serum samples were measured using commercially available enzyme immunoassay kits (Huakang Biotech, Shenzhen; Kehua Bio-Engineering, Shanghai, China) for the presence of HBsAg, antibody against HBsAg (anti-HBs), and anti-HBc. The HBsAg-positive sera were further tested for hepatitis B e antigen (HBeAg) and antibody against HBeAg by enzyme immunoassay kits (Huakang Biotech; Kehua Bio-Engineering). Quantitative levels of anti-HBs were tested with microparticle immunoassay (Architect system, Abbott, North Chicago) and expressed in international units. HBV DNA was quantitatively measured using real-time PCR assay (Shenyou Biotech, Shanghai, China), with the detection range 100–2×10^7^ IU/ml.

### Statistical analysis

Statistical analysis was performed with the SPSS software (SPSS Standard version 17.0, SPSS Inc., Chicago, IL). Continuous variables normally distributed were expressed as mean ± standard deviation and compared by *t*-test between two groups; quantitative data non-normally distributed were presented as median and interquartile range. Categorical variables were reported as number and percentage and compared by *χ* ^2^ test or Fisher's exact test where appropriate. Binary logistic regression analyses were further performed to determine the independent role of the feeding mode in the mother-to-child transmission of HBV or the immune response against HBV, after adjusting for the confounding effect of the other factors significantly different (with a P<0.20) between the two groups; the results were expressed by the adjusted odds ratios (aOR) with 95% confidence intervals (CI). A two-sided P value<0.05 is considered statistically significant.

## Results

### General characteristics of the study population

Totally, 546 children (2 pairs of twins) of 544 HBsAg-positive mothers were included in the study. The mothers were 23–48 years old (mean, 31.0±3.5), had no coinfection with hepatitis C virus and human immunodeficiency virus, and did not receive anti-HBV therapy before and during pregnancy. The children were at the age of 1–7 years (mean, 4.7±1.7). Of them, 137 (25.1%) were born to carrier mothers with HBeAg positive. All children had been vaccinated against hepatitis B and 291 (53.3%) received HBIG.

Of the children, 397 (72.7%) were breastfed and 149 (27.3%) others were formula-fed. Compared with those in formula-fed group, the mothers who breastfed their babies had similar ages and delivery modes (Table 1). However, the mothers' HBeAg-positive rate in formula-fed group was significantly higher than that in breastfed group (49.0% vs. 15.9%, *P*<0.001). This is possibly due to the notion that HBeAg-positive mothers are more contagious than HBeAg-negative carrier mothers. On the other hand, children's ages, gender ratios, and hepatitis B vaccine coverage and administration of HBIG were comparable between the two groups.

**Table 1:** General characteristic of children and their mothers in breast- and formula-fed groups.

Characteristic	Breastfed n = 397 (%)	Formula-fed n = 149 (%)	*P* value
Maternal age (years)	31.2±3.5	30.7±3.7	0.181
Mother with positive HBeAg	63 (15.9)	72* (49.0)	<0.001
Child's age (years)	4.8±1.7	4.5±1.7	0.058
Child's sex			
Male	215 (54.2)	81 (54.4)	0.966
Female	182 (45.8)	68 (45.6)	
Delivery			
Cesarean section	202 (50.9)	83 (55.7)	0.315
Vaginal delivery	195 (49.1)	66 (44.3)	
Timely first dose vaccine	359 (90.4)	130 (87.2)	0.279
Hepatitis B vaccine coverage	397 (100)	149 (100)	ND
HBIG	205 (51.6)	86 (57.7)	0.205

ND, not determined; HBIG, hepatitis B immune globulin; HBeAg, hepatitis B e antigen.

* Two pairs of twins were born to HBeAg-positive mothers in formula-fed group.

### HBV infection in breastfed and formula-fed children

Overall, of the total 546 children, 13 (2.4%) were chronically infected with HBV since positive results of HBsAg, anti-HBc, and HBeAg were observed in the children at the age of 1–7 years. Of the 13 children, 5 were administrated both HBIG and hepatitis B vaccine after birth, but the 8 others were only vaccinated against hepatitis B. Thus, the rate of chronic infection in the children with passive-active prophylaxis was 1.7% (5/291), which was lower than that (3.1%, 8/255) in the children with only active prophylaxis (*P* = 0.278). Importantly, chronic HBV infection occurred in 1.5% (6/397) of breastfed children and 4.7% (7/149) of formula-fed children respectively (Table 2); the difference was borderline statistical significance (*P* = 0.063). Additionally, the rate of self-resolved infection, presented as anti-HBc-positive but HBsAg-negative, was 2.3% in breastfed children, comparable to that (5.4%) in formula-fed children (*P* = 0.114).

**Table 2:** HBV serologic markers in breast- and formula-fed children of HBsAg-positive mothers.

	Breastfed n = 397 (%)	Formula-fed n = 149 (%)	*P* value
HBsAg+	6 (1.5)	7 (4.7)	0.063
Anti-HBc+/HBsAg−	9 (2.3)	8 (5.4)	0.114
Anti-HBs ≥10 mIU/ml	290 (73.0)	101 (67.8)	0.224

HBV, hepatitis B virus; HBsAg, hepatitis B surface antigen; anti-HBc, antibody against hepatitis B core antigen; anti-HBs, antibody against HBsAg; OR, odds ratio; CI, confidence interval.

It has been recognized that mother-to-child transmission of HBV is more likely to occur in infants of HBV-infected mothers with HBeAg positive and high HBV DNA levels [14], [15]. In accordance with this finding, we found that all 13 chronically infected children were born to HBeAg-positive mothers, while none of the children born to HBeAg-negative mothers was infected during follow-up. Thus, we further compared the infection rate in 137 children born to HBeAg positive mothers. Of them, 63 were breastfed and 74 were formula-fed; their mothers had similar HBV DNA levels (breastfed 2.32×10^6^ vs. formula-fed 2.47×10^6^ IU/ml, *P* = 0.613). Breast- and formula-fed infants had comparable prevalence of HBsAg (6/63, 9.5% vs. 7/74, 9.5%, *P* = 0.990) and of anti-HBc-positive/HBsAg-negative (7.9% vs. 10.8%, *P* = 0.567).

To further investigate whether breastfeeding may be an independent risk for the mother-to-child transmission of HBV, we performed two logistic regression analyses, one for the HBsAg positivity and the other for the prevalence of anti-HBc. As shown in Table 3, adjusting for the confounding effects of maternal HBeAg status and the child's age, breastfeeding was neither associated with chronic HBV infection (*P* = 0.995, aOR 0.99, 95%CI 0.28–3.58) or with the self-resolved infection (*P* = 0.301, aOR 0.57, 95%CI 0.20–1.65) in the children. In addition, we found that maternal HBeAg carriage was an independent risk for the mother-to-child transmission.

**Table 3:** Logistic regression analyses for the prevalence of HBsAg or anti-HBc in the children*.

	HBsAg+			anti-HBc+/HBsAg−		
	aOR	95%CI	*P* value	aOR	95%CI	*P* value
Breastfeeding	0.99	0.28–3.58	0.995	0.57	0.20–1.65	0.301
Maternal HBeAg carriage	1.69E8	0.00–∞	0.992	8.93	2.76–28.88	<0.001
Child's age	1.01	0.68–1.51	0.968	1.44	0.97–2.13	0.068

HBV, hepatitis B virus; HBsAg, hepatitis B surface antigen; anti-HBc, antibody against hepatitis B core antigen; aOR, adjusted odds ratio; CI, confidence interval.

* Including all the factors different (with a P<0.20) between the breastfed and formula-fed children except the mother's age, as which is not associated with the mother-to-child transmission of HBV [16].

### Anti-HBs in breastfed and formula-fed children

Of the 546 children who underwent regular immunoprophylaxis against hepatitis B, 391 (71.6%) showed anti-HBs ≥10 mIU/ml, which is in agreement with that of the children at the same age in a recent study [17]. As shown in Table 2, the positive rate of anti-HBs in breastfed children was comparable to that in formula-fed children (290/397, 73.0% vs. 101/149, 67.8%, *P* = 0.224). Furthermore, in the logistic regression analysis, we found that breastfeeding was independently associated with higher prevalence of anti-HBs in the children (*P* = 0.024, aOR 1.67, 95%CI 1.07–2.61).

## Discussion

In our study, the HBV infection rates were comparable in breastfed and formula-fed children of HBV-infected mothers after currently recommended immunoprophylaxis against hepatitis B, indicating that breastfeeding in high-risk children does not add any risk for mother-to-child transmission of HBV. In addition, breastfeeding may not interfere with humoral immune response to hepatitis B vaccine and HBIG. Therefore, we should not recommend HBV-infected mothers to formula-feed their children to prevent mother-to-child transmission of HBV.

Although HBV DNA may be detected in breast milk, especially in colostrum [6], in the present study, we observed that breastfed children had statistically comparable rate of HBV infection with that in formula-fed children (Table 2). However, the HBV infection rate in formula-fed children was higher than that in breastfed children (breastfed 4.7% vs. formula-fed 1.5%); the difference was likely due to the higher mothers' HBeAg-positive rate in formula-fed group than that in breast-fed group (49.0% vs. 15.9%), since children born to mothers with HBeAg positive are at a higher risk of being infected with HBV [14] and other general characteristics were comparable between the two groups (Table 1). Further analysis of the HBV infection in 137 children of HBeAg-positive mothers showed that breastfed and formula-fed children, whose mothers had similar HBV DNA levels, were at comparable risk of being infected with HBV. Additionally, logistic regression analyses demonstrated that breastfeeding does not pose additional risk for mother-to-child transmission of HBV (Table 3).

Before the availability of HBIG and hepatitis B vaccine, Beasley et al [10] reported a cohort of 92 breastfed babies and 55 non-breastfed babies, whose mothers were HBsAg-positive during and after delivery. When the infants were tested at 3 or more months old, with a mean follow-up period of 11 months, no significant difference was found in the acquisition rate of HBsAg (49% vs. 53%) or anti-HBs between the two groups. These results demonstrated no correlation between breastfeeding and the development of HBV infection in children of carrier mothers. It may be related to the finding that the exposure to HBV associated with breast milk is negligible compared to that from maternal blood and other body fluids during delivery or labor, and therefore children rarely acquire HBV infection through breast milk with weaker infectiousness [2]. With the availability of HBIG and hepatitis B vaccine, a study conducted in United States found comparable rates of HBV transmission in breast- and formula-fed children (0% vs. 3%) at the age of 9–15 months, indicating that breastfeeding does not add any risk for mother-to-child transmission of HBV [11]. Our data in the present study are in accordance with these reported results.

Although all the children in our study were vaccinated against hepatitis B, only 53.3% were administered HBIG, considerably lower than the rate in the developed countries [18], [19]. The substantial gaps in the immunoprophylaxis of the infants between the recommendations and routine practices were attributable to the unavailability of HBIG, incomplete knowledge on the prophylaxis of the health care providers, and low prenatal HBsAg screening rate, particularly in rural areas [20]. Additionally, we observed that the children who received HBIG had a lower rate of chronic infection than those who did not receive HBIG (1.7% vs. 3.1%). Therefore, full adherence to the prophylaxis recommendations is a more important point than whether the mother breastfeeds or not in reducing the mother-to-child transmission.

Recently, the efficacy of oral antiviral therapy in HBV-infected pregnant women for preventing mother-to-child transmission has been investigated [21]–[24]. There appears to be a shift towards implementing selective antiviral prophylaxis in the third trimester for pregnant women with higher viral loads. However, some studies were non-prospective or recruited only limited number of study subjects [21], [22]. The data in a randomized controlled trial were difficult to interpret because as high as 39% of the infants who received standard administration of HBIG and hepatitis B vaccine after birth were infected at age of 12 months [23]. So far, there is no consensus or guideline on the therapy choice regarding maternal viral loads and nucleoside/nucleotide analogues [25], [26], and more convincing prospective controlled trials are required to demonstrate the benefit of therapy in reducing the likelihood of maternal-infant transmission, when the newborn infants are administered both HBIG and vaccination on time [27], [28]. Thus, the pregnant women in the present study had not been advised to receive antiviral therapy.

The main limitation in our study was its non-randomized design. The children's feeding modes were not randomly assigned and the HBeAg-positive rate was much higher in the mothers of the formula-fed children; this may contribute to the relatively lower prevalences of HBsAg and anti-HBc in breastfed children (Table 2). To overcome the bias due to maternal HBeAg status and other different characteristics between the breastfed and formula-fed children, we further performed logistic regression analyses to evaluate the impact of breastfeeding on the mother-to-child transmission. Our results indicated that breastfeeding was not associated with the HBV infection in the children (Table 3). There may be other potential bias or even unknown different factors in the non-randomized study. Nevertheless, the comparable prevalence of HBsAg in formula-fed and breastfed children indicates that breastfeeding does not increase maternal-infant transmission of HBV. Additionally, we did not consider it ethically justified to decide the children's feeding modes.

In view of these results, we conclude that breastfeeding is not a risk factor for mother-to-child transmission of HBV after the recommended prophylaxis is implemented. Therefore, regardless of mothers' status of HBeAg, clinicians should encourage HBV-infected mothers to breastfeed their infants with available immunoprophylaxis against hepatitis B.
